# Insights gained from single-cell analysis of chimeric antigen receptor T-cell immunotherapy in cancer

**DOI:** 10.1186/s40779-023-00486-4

**Published:** 2023-11-08

**Authors:** Lu Tang, Zhong-Pei Huang, Heng Mei, Yu Hu

**Affiliations:** 1 Institute of Hematology, Union Hospital, Tongji Medical College, Huazhong University of Science and Technology, Wuhan, 430022 China; 2 Hubei Clinical Medical Center of Cell Therapy for Neoplastic Disease, Wuhan, 430022 China; 3 Key Laboratory of Biological Targeted Therapy, The Ministry of Education, Wuhan, 430022 China; 4 Hubei Key Laboratory of Biological Targeted Therapy, Union Hospital, Tongji Medical College, Huazhong University of Science and Technology, Wuhan, 430022 China

**Keywords:** Single-cell sequencing, Cancer immunotherapy, CAR-T therapy, Cell heterogeneity, Trajectory inference, Tumor microenvironment

## Abstract

Advances in chimeric antigen receptor (CAR)-T cell therapy have significantly improved clinical outcomes of patients with relapsed or refractory hematologic malignancies. However, progress is still hindered as clinical benefit is only available for a fraction of patients. A lack of understanding of CAR-T cell behaviors in vivo at the single-cell level impedes their more extensive application in clinical practice. Mounting evidence suggests that single-cell sequencing techniques can help perfect the receptor design, guide gene-based T cell modification, and optimize the CAR-T manufacturing conditions, and all of them are essential for long-term immunosurveillance and more favorable clinical outcomes. The information generated by employing these methods also potentially informs our understanding of the numerous complex factors that dictate therapeutic efficacy and toxicities. In this review, we discuss the reasons why CAR-T immunotherapy fails in clinical practice and what this field has learned since the milestone of single-cell sequencing technologies. We further outline recent advances in the application of single-cell analyses in CAR-T immunotherapy. Specifically, we provide an overview of single-cell studies focusing on target antigens, CAR-transgene integration, and preclinical research and clinical applications, and then discuss how it will affect the future of CAR-T cell therapy.

## Background

Currently, the primary standard treatments for most malignancies are chemotherapy, radiation therapy, and, in the case of solid tumors, surgery. With the development of immune-based treatments, some therapies, such as cell therapy and immune checkpoint inhibitors, are promising for patients with refractory or relapsed cancer patients [1–5]. The tremendous clinical successes of chimeric antigen receptor (CAR)-T cell therapy in hematologic malignancies have led to an exponential growth in research within the field and revolutionized anticancer immunotherapy [6–9]. The CAR is an engineered molecule that includes a single chain fragment variable (scFv) against a specific antigen. CAR-T cells work by recognizing and binding to the scFv target on the malignant cells. The U.S. Food and Drug Administration (FDA) has approved six kinds of CAR-T cell products since 2017 [10–18] and catapulted this field into an era of fast-paced and innovative research. As more patients are being treated and more extended follow-up data are becoming available, it has been realized that only a fraction of patients received clinical benefit from these CAR-T cell therapies [19, 20]. Moreover, many clinical trials have revealed frequent and various adverse events, exposing the limitations of CAR-T cell therapy. Although traditional techniques can enable high-throughput and quantitative profiling of genomic, transcriptomic and proteomic signatures, for bulk populations, cells must be placed in groups on the basis of the expression levels of established surface markers, which can potentially introduce bias into the results [21–24]. Thus far, progress in addressing therapeutic failures has been limited due to the inability to fully characterize CAR-T cell behaviors in vivo at the single-cell level, posing a major hurdle to the extensive application of CAR-T immunotherapy.

The emergence of single-cell sequencing has already led to substantial advances in the identification of novel biomarkers, cellular phenotypes and therapeutic targets, many of which would have been undetectable by bulk-sequencing approaches [25–27]. Single-cell sequencing offers high resolution and is best suited to studying the properties of immune cells, including diverse developmental lineages, antigen specificity, phenotypic plasticity, and adaptability to various microenvironments. This powerful tool makes it possible for us to take an in-depth look into the behaviors and fate of CAR-T cells [24, 27–32]. Accumulating evidence shows that single-cell methods can help researchers work out better receptor design, guide gene-based T cell modification and optimize manufacturing conditions, which are essential for long-term immunosurveillance and more favorable therapeutic outcomes [33–39]. Furthermore, single-cell sequencing contributes to clinical monitoring by enabling the prediction of therapeutic efficacy and toxicities, which facilitates individualized treatment and greatly raises the prospects of CAR-T therapy [40, 41]. In this review, we discuss why CAR-T cell immunotherapy fails in clinical practice and what this field has learned since the milestone of single-cell sequencing technologies. We further outline recent advances in the application of single-cell sequencing to CAR-T immunotherapy. Specifically, we present an overview of the single-cell studies focusing on target antigens, CAR-transgene integration, and following preclinical research and clinical applications, and then discuss how they will affect the future of CAR-T cell therapy.

## What impedes CAR-T function?

### Cancer resistance and relapse

Therapeutic failure due to cancer resistance and relapse remains one of the major challenges hindering the use of CAR-T cell therapy [20, 42–46]. The common mechanism associated with relapse after CAR-T cell therapy is loss of the target antigen, which makes the molecule unrecognizable by CAR-T cells, and the loss of targets may be caused by negative antigens or antigen deficiency [9, 47–55]. Genetic mutations in CD19 developed in the majority of resistant tumor cells and irreversible loss of heterozygosity at the time of CD19-negative relapse [47], indicating that mutation of target antigen under selective pressure is one of the crucial mechanisms underlying antigen loss. CAR-T cells also induce reversible antigen loss through trogocytosis, an active process in which the target antigen is transferred to T cells, thereby reducing target density on cancer cells and decreasing T cell activity by promoting fratricidal T cell killing and inducing T cell exhaustion [55]. In addition, the unintentional introduction of the CAR gene into a single leukemic B cell during T cell manufacturing resulted in CAR-T cell escape. Its product was bound in cis to the CD19 epitope on the surface of leukemic cells, masking it from recognition by CAR-T cells and thereby conferring resistance [46].

However, not all patients suffered an antigen-negative relapse, suggesting that additional factors may contribute to therapeutic failure [43, 56–71]. CAR-T cells are typically produced from lymphocytes that are derived from autologously collected apheresis samples. Therefore, the composition of CAR-T cells is unique to individual patient, and is tremendously heterogeneous. CAR-T cells from responders were enriched in memory-related genes, interleukin-6 (IL-6) and signal transducer and activator of transcription 3 (STAT3) signatures, whereas CAR-T cells from non-responders up-regulated programs involved in effector differentiation, glycolysis, exhaustion, and apoptosis [43]. NR4A transcription factors play an essential role in the cell-intrinsic program of T cell hyporesponsiveness, thereby limiting CAR-T cell function [60]. Programmed death-1 (PD-1)-mediated T cell exhaustion also affected CAR-T cells, suggesting that PD-1/PD-L1 blockade may be an effective strategy to improve the efficacy of CAR-T cell therapies [72–74].

Tumor endogenous factors and the tumor microenvironment (TME) are also thought to play a pivotal role in the therapeutic response to CAR-T therapy. Several researchers have conducted integrated drug profiling and CRISPR (clustered regularly interspaced short palindromic repeats) screening to identify essential pathways responsible for CAR-T cell cytotoxicity [56, 57]. Impaired death receptor signaling in tumor cells causes failed cytotoxicity and drives CD19-targeted CAR-T cell dysfunction, identifying a novel mechanism culpable for the antigen-independent resistance. More importantly, highly-immunosuppressive solid malignancies, like glioblastoma or pancreatic cancer, may also generate resistance to CAR-T therapy. Immunosuppressive cells and cytokines, such as regulatory T cells, myeloid-derived suppressor cells, and transforming growth factor β, can inhibit the proliferation and effector functions of CAR-T cells [75–78]. The lack of chemokines leads to an impaired chemotactic ability of CAR-T cells. In addition, the abnormal vasculature and cancer-associated fibroblasts make it difficult for CAR-T cells to migrate to the tumor [79, 80].

### Toxicity

Toxicities appear to be severe effects of CAR-T therapy, such as cytokine release syndrome (CRS) and immune effector cell-associated neurotoxicity syndrome (ICANS) [81–88]. The cytotoxicity of some CAR-T cells is not highly tumor-specific and can cause damage to normal tissues. Therefore, it is imperative to comprehensively characterize CAR-T cells and elucidate the mechanisms responsible for successful CAR-T cell immune responses and reduced toxicities. This remains a critical task for further refinement of CAR-T cell-based immunotherapy. CRS is the most frequently observed life-threatening adverse effect of CAR-T cell therapy. CAR-T cell-mediated cancer clearance triggers the systemic inflammatory response with elevated IL-6 levels, which is the hallmark of CRS [81]. Therefore, clinically, the primary management to overcome CRS is to break the cytokine feedback loop by treating it with an IL-6 receptor monoclonal antibody [89]. Corticosteroids combined with tocilizumab are also used to reduce inflammation and vasogenic edema in the brain and reduce the potentially fatal severity of the disease [90, 91]. Notably, in the pathophysiological process of CRS, endogenous immune cells such as monocytes, macrophages, and dendritic cells are also involved in the synthesis and release of various cytokines and are responsible for the symptoms of CRS [83, 92]. Neurotoxicity is associated with CRS and manifests as transient loss of working memory, delirium, seizures, and, in rare cases, acute cerebral edema [87]. Clinically, ICANS can present as early as the day after or as late as the fourth week after CAR-T infusion [88]. The development of neurotoxicity is associated with a higher pretreatment disease burden, higher peak CAR-T cell expansion, and earlier and higher levels of proinflammatory cytokines in blood and cerebrospinal fluid [88]. Gust et al. also reported that endothelial dysfunction and increased blood–brain barrier permeability may allow inflammatory cytokines and immune cells to enter the central nervous system and contribute to neurological inflammation [93, 94].

## The limitations of traditional experimental techniques

During the growth and development of organisms, their transcriptome information varies with different cell types, external environments and internal regulatory factors. Cellular heterogeneity is also a universal feature of biological tissues and systems. Conventional next-generation sequencing can be used to detect the genomic and transcriptomic information of a cell population, such as a large number of cells, animal and plant tissues, or even an organism as a whole. This approach can yield a myriad of genomic or transcriptome data, but obscures the gene expression patterns of individual cells and is not capable of revealing cellular heterogeneity. To distinguish cells in a bulk cell population, the cells must be categorized on the basis of the expression levels of established surface markers, which might potentially introduce bias. Since human tumors are intricate multicellular ecosystems composed of diverse cells, including subsets of cancer, immune, and stromal cells, their comprehensive characterization cannot be achieved by bulk-omics methods. Single-cell sequencing offers high resolution of many aspects of immune cells, including diverse developmental lineages, antigen specificity, phenotypic plasticity, and adaptability to various microenvironments [95]. Despite the large amount of data generated by traditional experimental techniques, the single-cell omics profiles related to therapeutic responses and adverse events remain unknown. Comprehensive characterization of CAR-T cell behaviors and host immune states at the single-cell level will be more helpful for overcoming therapeutic failures.

## Current advances in single-cell sequencing technologies

With remarkable technological advances and exponentially growing cellular throughput, single-cell sequencing has become feasible and covered multiple aspects of omics, including genomic, transcriptomic, epigenomic, and proteomic characteristics of individual cells, as well as any combination thereof [96–117]. Moreover, in combination with the improvements in spatial techniques, single-cell sequencing is also helpful for the dissection of immune cell communication networks at the systemic level [118–121]. A wide variety of screening platforms have been established for interrogating molecular assays at the single-cell level to explore the heterogeneity of tumors and the clonal evolution of highly complex tissues. The 10 × Genomics and BD Rhapsody™ Single-Cell Analysis System have been commercially available and extensively employed for single-cell analysis since 2017 [122]. In addition to these commercial platforms, intensive research has led to the development of numerous methods for more in-depth characterization of single cells. In recent years, scientists and research institutions have established many single-cell databases to help solve various medical problems. These databases collect large data sets and provide researchers with more user-friendly platforms. They cover a wide range of species, organs, tissues, cell lines, diseases and data types of single-cell sequencing. The integrative gene expression data can help us to identify new cell types or sub-regions in organs [123], analyze the intercellular interactions in TME [124], and understand the mechanisms of side effects of the therapies [125]. They can even successfully handle complex analyses of single-cell sequencing data. As a result, more researchers are beginning to use these portals to extract more information and increase the richness and credibility of their studies. Herein, we focus on the technological advances and applications of single-cell sequencing (Table 1).

**Table 1:** Summary of techniques of single cell sequencing

Omics	Technique	Application	Limitation
Genome	SNS [105]	Detect DNA mutations including CNVs, SVs, SNVs in cancer cells, contributing to knowing dynamics of tumorigenesis, heterogeneous of tumors and identifying specific cancer clones	Low throughput
	SCI-seq [106]		Currently limited to CNV detection
	SCANPY [107]		Python-based rather than R-based frameworks
	SMOOTH-seq [108]		Low throughput
Epigenome	scATAC-seq [109]	Detect the open status of chromatins; Link regulatory elements to their target genes and discover in vivo correlates of heterogeneity in accessibility within cell types; Explain heterogeneity in the shift of cell states and epigenetic mechanisms for CAR-T exhaustion, differentiation and resistance	Remain challenges in detection of large-scale SVs and haplotype phasing Speculate TF binding from motif or chromatin profiling data indirectly
	sciATAC-seq [110]		Speculate TF binding from motif or chromatin profiling data indirectly
	scNanoATAC-seq [111]		Expensive under current sequencing depth (less than 2.5 dollars per cell) Speculate TF binding from motif or chromatin profiling data indirectly
	scTHS-seq [126]	Improve coverage of highly cell specific distal enhancers compared with scATAC-seq	High cost
	scDrop-CHIP [112]	Learn heterogeneity of tumors from aspects of histone modification and transfer factor binding directly, offer the opportunity to predict sensitivity to therapeutic agents [127]	Non-duplicated reads are low (less than 1000)
	scCUT&Tag [113, 114]		High cost
	Spatial-CUT&Tag [115]		High cost
	scBS-seq [116]	Detect DNA methylation to study cell development and connections between methylation and diseases	Very low cell throughput
	scCGI-seq [117]		
	snmC-seq [128] snmC-seq2 [129]		High cost
	sci-MET [130]		The coverage of methylation is not enough
	scRRBS [131]		Very low cell throughput Not full-length genome
	scHi-C-seq [83]	Specific for chromosome conformation	Very low cell throughput
Transcriptome	SMART-seq SMART-seq2 [132] SMART-seq3 [133]	Suitable for samples with small numbers of cells; Find rare fragments in mRNA by its whole mRNA sequencing	Low throughput Long experimental period
	CEL-seq [134] CEL-seq2 [135]	Identify cell types, discover tumor cells and TME, explain treatment outcomes and mechanism of diseases from clonal dynamics and other transcriptional profiles; MARS-seq2.0 enables intuitive approaches for depletion or enrichment of cell populations	Low throughput 3′-end counting, not full-length
	MARS-seq [136]/MARS-seq2.0 [137]		3′-end counting, not full-length
	Drop-seq [102]		
	InDrop [138]		
	Seq-Well [139]		
	MULTI-seq [140]		
	Microwell-seq [141] /Microwell-seq2.0 [142]	It’s cost-efficient, allowing for the mapping of cell atlas [141, 142]	Not full-length
	10 × Genomics [143, 144]	Beside applications above, they can acquire sequencing of TCR & BCR beside gene transcripts as well, which are helpful for immune cell phenotype and associated cancers	High cost
	BD Rhapsody [145]		
	SPLiT-seq [146]	It allows super high-throughput sequencing, and support to draw comprehensive atlas of cells or find rare cell clusters	3′-end counting, not full-length
	sci-RNA-seq [147] sci-RNA-seq3 [148]		Limited to detect exon High cost
	sci-Plex [149]	sci-Plex scales to thousands of samples, enables high-throughput sequencing and multiplex chemical transcriptomics at single-cell level. It’s a cost-effective and scalable method, allowing large-scale drug screening	Only affix barcodes to nuclei of cells, which means detecting mRNA in nuclei alone
	scISOr-seq [150]	Using the third-generation sequencing platform to get full-length transcripts	High cost Low throughput High error rate
	ScNaUmi-seq [151]		
	R2C2 [152]		
Proteome	High-resolution microscopy [153]	Quantify proteins in single cells; Biomarker detection	The number of screened proteins is small High false positive rate
	Flow cytometry [154]		
	Immunohistochemistry [155]		
	CyTOF [156]	Quantify multiple proteins in single cells; Immune profiling; Biomarker discovery	Limited in the number of parameters cells per sample they can simultaneously assess High cost
	BD Abseq [157] BioLegend TotalSeq [158]		High cost
	CITE-seq, REAP-seq, SCITO-seq ^[159–162]^	Single-cell sequencing is performed simultaneously at the transcriptome and proteome levels	

*SNS* single nuclear sequencing, *SCI-seq* single-cell combinatorial indexed sequencing, *SMOOTH-seq* single-molecule real-time sequencing of long fragments amplified through transposon insertion, *scATAC-seq* single-cell assay for transposase-accessible chromatin using sequencing, *sciATAC-seq* single-cell combinatorial indexing assay for transposase-accessible chromatin using sequencing, *CNVs* copy number variations, *SVs* structure variations, *SNVs* single nucleotide variants, *TF* transcription factors, *scNanoATAC-seq* single-cell assay for transposase-accessible chromatin on Nanopore sequencing platform, *scTHS-seq* single-cell transposome hypersensitive site sequencing, *scChIP-seq* single-cell chromatin immunoprecipitation followed by sequencing, *TCR* T cell receptor, *BCR* B cell receptor, *scCUT&Tag* single-cell Cleavage Under Targets and Tagmentation, *scBS-seq* single-cell bisulfite sequencing, *scCGI-seq* genome-wide CpG island methylation sequencing for single cells, *snmC-seq* single nucleus methylcytosine sequencing, *sci-MET* single-cell combinatorial indexing for methylation analysis, *scRRBS* single-cell reduced representation bisulfite sequencing, *scHi-C-seq* single-cell Hi-C method for chromosome conformation, *SMART-seq* switching mechanism at 5′ end of the RNA transcript sequencing, *CEL-seq* cell expression by linear amplification and sequencing, *MARS-seq* massively parallel RNA single-cell sequencing, *MULTI-seq* multiplexing using lipid-tagged indices for single-cell and single-nucleus RNA sequencing, *SPLiT-seq* split-pool ligation-based transcriptome sequencing, *sci-RNA-seq* single-cell combinatorial indexing RNA sequencing, *sci-Plex* single-cell combinatorial indexing for multiplex transcriptomics, *scISOr-seq* single-cell isoform RNA sequencing, *ScNaUmi-seq* single-cell Nanopore sequencing with UMIs, *R2C2* rolling circle amplification to concatemeric consensus, *CyTOF* mass cytometry by time-of-flight, *CITE-seq* cellular indexing of transcriptomes and epitopes by sequencing, *REAP-seq* RNA expression and protein sequencing, *SCITO-seq* single-cell combinatorial indexed cytometry sequencing

### Genome

Single-cell genome sequencing allows us to get better understanding with genetic heterogeneity and identify the driving mutations that are responsible for cancer progression and metastasis [105, 163, 164]. An in-depth insight into the clonal dynamics of cancer cells is vital to dissecting the subpopulations of the cancer cells that could then be profiled to identify novel tumor-specific antigens (TSAs) [24]. A great many methods are available for the analysis of single-cell gene expression profiles, such as those conducted by single nucleus sequencing (SNS), single-cell combinatorial indexed sequencing (SCI-seq), and SCANPY [105, 107, 165, 166]. Moreover, single-molecule real-time sequencing of long fragments amplified through transposon insertion (SMOOTH-seq), a novel single-cell whole genome sequencing (scWGS) technique based on the third-generation platform, has also been developed [108]. These approaches can acquire genomic data, detect copy number variations (CNVs), structural variations (SVs), and single nucleotide variants (SNVs) in human cancer cells in particular, and facilitate information mining for these DNA mutation-related diseases. In the meantime, the scWGS has helped researchers gain unprecedented insights into the clonal dynamics that occur during tumorigenesis and during normal hematopoiesis [163, 167, 168]. The scWGS can be used to characterize mutational profiles of highly heterogeneous tumors at the single-cell resolution scale, which is particularly useful in determining which clones are highly vulnerable or resistant to CAR-T cell-mediated cytotoxicity [24]. Moreover, single-cell genomics technologies afford the possibility to fully annotate the antigen expression on all human tissues and cancer cells, which may be helpful in the de-risking of potential targets and the identification of optimal antigens for CAR-T therapy [169].

### Epigenome

Epigenetic regulation involves chromosome conformation, chromosome accessibility, DNA and RNA methylation, histone modification, and the regulation of non-coding RNA [95]. The three-dimensional structure models of the whole genome exhibit a radially-organized architecture of chromosome-based compartments with distinctive epigenomic features [170]. The single-cell Hi-C platforms have been established to provide chromatin architecture mapping in individual cells, which allows the measurement of the spatial proximity between transcription regulatory elements in a cell type-dependent manner [171]. The single-cell assay for transposase accessible chromatin sequencing (scATAC-seq) enables the cutting of open chromatins through Tn5 transposase, and these fragments will be further amplified, thus providing epigenetic information through sequencing [109]. It could be used for the measurement of open chromatin regions of DNA and to determine which genes are accessible for transcription. However, this technology is limited to detecting large SVs (including insertions, deletions, duplications, inversions, and translocations) and haplotype phasing [111]. To address the challenge, Tang et al. developed single-cell assay for transposase-accessible chromatin on Nanopore sequencing platform (scNanoATAC-seq) for single-molecule long-read sequencing. Currently, scATAC-seq is being extensively and intensively used and holds great promise to explain the heterogeneity in the shift of immune cell status [172–176]. Besides, single-cell chromatin immunoprecipitation followed by sequencing (scChIP-seq) can be used for detecting histone modification and binding of transcription factor [112–114]. The spatial Cleavage Under Targets and Tagmentation (CUT&Tag) has been established for the spatial-resolution genome-wide profiling of histone modifications, opening new avenues for the study of epigenetic regulation, cell function, and fate decision in tumor pathogenesis and therapeutic intervention [115]. The global methylation status of CAR-T cells may be related to the efficacy of the treatment [24, 62, 177, 178]. Several methods have been developed for the single-cell profiles of DNA and RNA methylation, such as single-cell bisulfite sequencing (scBS-seq), single nucleus methylcytosine sequencing (snmC-seq), single-cell combinatorial indexing for methylation analysis (sci-MET) and single-cell reduced representation bisulfite sequencing (scRRBS), et al. [128, 130, 179, 180]. Therefore, single-cell resolution information about the epigenetic programming of the CAR-T cells may provide a method to further improve the performance of CAR-T cells [172].

### Transcriptome

scRNA-seq is a powerful technique used in molecular biology and genomics to study gene expression at the single-cell level and is the most popular method among single-cell technologies. scRNA-seq can help to identify differentially-expressed genes, perform clustering analysis to identify cell subpopulations and construct cell lineage trees to understand the developmental trajectories of different cell types [97, 132, 134]. Smart-seq was developed in 2013 and was one of the first scRNA-seq methods to enable full-length transcript sequencing from single cells [181]. Smart-seq2 was developed in 2014 as an improved version of smart-seq [182]. The sensitivity of smart-seq3 has been greatly increased compared to smart-seq2, typically detecting thousands of more transcripts per cell [133]. Cell expression by linear amplification and sequencing (CEL-seq) and CEL-seq2 involve barcoding individual cells with unique molecular identifiers (UMIs) and capturing mRNA transcripts using oligo-dT primers [134, 135]. Massively parallel RNA single-cell sequencing (MARS-seq) uses a microfluidic device to separate cells into nanoliter-sized droplets, enabling high-throughput processing of thousands of cells at the same time [137]. Despite high sequencing depth per single cell, these methods are limited by low throughput. Droplet-based microfluidics is one of the most challenging candidates for the capture and processing of thousands of individual cells in parallel for whole-transcriptome analysis. It enables the assessment of large numbers of cells at a relatively low cost [138, 183]. Like nanowell-based implementations, the Seq-Well places single cells and barcoded poly (dT) mRNA capture beads in a polydimethylsiloxane array of ~ 86,000 subnanoliter wells. It enables high-throughput scRNA-seq of low-input samples [139, 144]. In addition to two well-known commercial platforms, 10 × Genomics (droplet-based) and BD Rhapsody (nanowell-based), other high-throughput scRNA-seq methods have also been developed, such as Multiplexing using lipid-tagged indices for single-cell and single-nucleus RNA sequencing (MULTI-seq) [140] and Split-pool ligation-based transcriptome sequencing (SPLiT-seq) [146]. With the development of technologies that integrate whole-transcriptome sequencing and targeted gene enrichment to capture T cell receptor (TCR) and B cell receptor (BCR) repertoires, scRNA-seq has also been used to gain knowledge about the specific immune responses that occur during disease progression [184, 185]. Cell subsets originating from a single cell can be traced by sequencing TCR or BCR RNA transcripts, facilitating the study of clonal dynamics. Likewise, the direct relationship between clonotypes and phenotypes can be addressed. The 5′-ends of mRNA are labeled with barcoded template-switch-oligos and PCR (Polymerase Chain Reaction) is performed with TCR- or BCR-specific primers in the technique developed by 10 × Genomics. This method is highly efficient in capturing the fragment containing the V-D-J segments because these segments are located at the 5′ end of the mRNA. In this way, it's possible to study the clonal dynamics of CAR-T cells [186–192]. Several studies have been conducted to make clinic-relevant statements about the transcriptional profiles of CAR-T cells that are related to treatment outcomes, whether favorable or poor [41, 193]. More importantly, scRNA-seq can be an effective tool to study the interactions that occur within the TME between a wide variety of endogenous immune cell types and CAR-T cells [194].

### Proteome

It’s also informative to explore heterogeneity at the protein level because of its direct regulation of cellular function and transcriptome can’t reflect cell functions completely indeed. Proteomic sequencing as a whole is hindered by a lack of direct amplification of proteins due to their complex secondary and tertiary structures [195]. Conventional approaches like high-resolution microscopy, flow cytometry and immunohistochemistry allow for the quantification of proteins at the resolution of a single cell [24]. These methods can be used to track the location and expansion of CAR-T cells after cell infusion. However, these methods are limited by the number of proteins that can be screened at the same time. The mass cytometry by time-of-flight (CyTOF) has been widely applied in immune characterization, the discovery of biomarkers, and studies of therapeutic effects [196–205]. The main advantage of CyTOF over traditional flow cytometry is the capability to combine multiple antibody specificities in a single sample with negligible spillover between the channels [206, 207]. CyTOF has been used to assess the expression levels of surface or intracellular proteins that are associated with T cell function, helping to determine the specific CD4^+^ CAR-T subpopulations that have been linked to clinical outcomes [208]. Currently, fluorescent tags and heavy metal isotopes are conjugated to antibodies to evaluate protein levels, and this approach is constrained by the number of parameters per sample that can be assessed simultaneously [195, 196, 202]. In addition, the Abseq utilizes the conjugation of antibodies to the unique DNA barcodes that can be read with the microfluidic barcoding and DNA sequencing, and it has extended the number of proteins that can be measured up to the hundreds [159, 209, 210]. Several single-cell proteomics methods have been developed for use alongside scRNA-seq, such as cellular indexing of transcriptomes and epitopes by sequencing (CITE-seq), RNA expression and protein sequencing (REAP-seq) assay, and single-cell combinatorial indexed cytometry sequencing (SCITO-seq) [159–162]. Although these methods can theoretically be used for quantitative detection of intracellular proteins, they are restricted to the assessment of cell surface markers. Taken together, single-cell proteomic profiling combined with scRNA-seq allows for high-throughput monitoring of single-cell phenotypes [35, 211–214]. Therefore, it could provide a much higher resolution in defining specific CAR-T cell subsets associated with clinical outcomes [40, 215, 216].

## Overview of single-cell sequencing applied in CAR-T cell studies

The study of CAR-T cell therapy is dependent on various pre-clinical models that can reconstruct the interactions between cancer cells and immune cells and evaluate the therapeutic efficacy. Many fundamental questions need to be addressed at each stage of CAR-T cell therapy. How does the heterogeneity of T cells affect CAR-T cell therapy? To what extent is this heterogeneity modulated by CAR transduction and cell manufacturing? Are there predominant CAR-T cell subpopulations that are correlated with successful CAR-T cell therapies? Is it possible to predict clinical responses and adverse events of CAR-T cell therapy based on single-cell signatures? Accounting for cell heterogeneity and lineage tracing, single-cell sequencing has provided new insights at each and all stages of CAR-T cell therapy (Table 2) [16, 35, 216–227].

**Table 2:** Overview of representative studies of single-cell sequencing in research of CAR-T Cell Studies

Disease	Sample	Method	Data and platform	Key points	Reference
Pan-cancer	18 tissues and organs from healthy human	scRNA-seq	Public data from GEO	Develop a comprehensive single-cell atlas for target antigens of CAR therapy in normal tissues and organs, which helps to capture antigen-expressing rare cell types missed in the assessment of bulk tissues	[216]
Pan-cancer	Adult cell from HCL and AHCA	scRNA-seq	Two independent cohorts based on Microwell-seq and 10 × Genomics	Report a potential on-target, off-tumor toxicity landscape for CAR targets across a wide range of tissues Develop a user-friendly data portal, CAR target gene toxicity at single-cell level (CARTSC)	[217]
AML	Single cells from 15 individuals with AML and tissue from 9 healthy individuals	scRNA-seq	Publicly available scRNA-seq data	Identify CSF1R and CD86 as targets for CAR-T cell therapy in AML Extensive in vitro and in vivo validation revealed broad expression on AML blasts, strong and durable treatment responses of newly developed CAR-T cells in vitro and in vivo and minimal toxicities toward relevant healthy cells and tissue	[221]
B-NHL	Differently prepared CAR-T cells, infusion products, PBMCs from patients	scRNA-seq	10 × Genomics Illumina	The electroporation method resulted in a high percentage of memory T cells in infusion products, and PD-1 interference enhanced antitumor immune functions, further validating the advantages of non-viral, PD-1-integrated CAR-T cell	[218]
–	CAR-T cells	scATAC-seq	10 × Genomics Illumina NextSeq 550	Develop a method called EpiVIA for the joint profiling of the chromatin accessibility and lentiviral integration site analysis at the population and single-cell levels especially for CAR-T	[222]
LBCL	CAR-T cells	scRNA-seq	10 × Genomics Illumina HiSeq	Transcriptional signatures are related to costimulatory domains and signaling domains included in CARs uniquely shape the transcriptional programs of T cells	[219]
R/R B-ALL	Infusion products	scRNA-seq, CITE-seq	scFTD-seq Illumina HiSeq4000	Unveil heterogeneities of donor and patient CAR-T cells and provide mechanistic basis for ameliorating clinical outcomes and developing next-generation "off-shelf" allogeneic products	[35]
–	CAR-T cells	scRNA-seq, scCAR-seq	10 × Genomics Illumina NovaSeq	Generate a library of 180 unique CAR variants genomically integrated into primary human T cells by CRISPR-Cas9 Identify several variants with tumor killing properties and T cell phenotypes markedly different from standard CARs	[220]
–	CAR-T cells	Single-cell, 16-plex cytokine profiling	Single-cell barcode chip	Reveal a diverse landscape of immune effector response of CD19 CAR-T cells to antigen-specific challenge Significant subsets of stimulated CAR-T cells exhibit high polyfunctionality with a dominant antitumor effector cytokine profile	[223]
B cell malignancies	Pre-manufacture T cells from patients	scATAC-seq CITE-seq	10 × Genomics Illumina Nova-Seq 6000	Chronic interferon signaling regulated by IRF7 was associated with poor CAR-T cell persistence across T cell subsets, and the TCF7 regulon not only associates with the favorable naive T cell state, but is maintained in effector T cells among patients with long-term CAR-T cell persistence	[214]
B cell lymphoma	BM cells from one tibia and one femur per mouse	scRNA-seq	10 × Genomics Illumina HiSeq × 10	Antitumor activity mediated by CAR-T cells largely relies on cellular cross-talk within the TME. Mechanistically, IFN-γ produced by CAR-T cells and sensed by the host was essential to boost the cytotoxic potential of CAR-T cells and of host NK and T cells. CAR4 and CAR8 T cells exhibited complementary functions, being more efficient at immune activation and tumor killing, respectively	[194]
B-ALL	Pre-infusion products and post-infusion CD19-CAR-T cells from blood and bone marrow samples	scRNA-seq scTCR-seq	10 × Genomics Illumina NovaSeq	Pre- and post-infusion CAR-T cells have distinct gene-expression profiles. Pseudotime identifies two distinct trajectories for post-infusion CAR-T cell differentiation: the first trajectory involves effector differentiation characterized by the expression of conventional cytotoxic genes; the other trajectory indicates the rapid development of these same exhaustion and cell death signatures soon after infusion	[187]
B cell lymphoma	Pre-treatment and post-treatment PBMCs, infusion products	scRNA-seq scTCR-seq	10 × Genomics Illumina NovaSeq S4	Cellular dynamics of response differs between the two products: tisa-cel responses were associated with striking expansion of rare CD8^+^ central-memory-like populations from the IPs, whereas axi-cel treatment revealed less shifting of T cell lineages between IPs and post-treatment. CAR-Treg cells can suppress conventional CAR-T activity and thus facilitate relapse	[224]
CLL	PBMCs before manufacturing, pre-infusion products and post-infusion CAR-T cells	scRNA-seq scTCR-seq CyTOF CITE-seq	10 × Genomics Illumina NextSeq 550 Fluidigm Helios mass cytometer	Long-persisting CD4^+^ CAR-T cells exhibited cytotoxic characteristics along with ongoing functional activation and proliferation	[191]
B cell malignancies	CD8^+^ CAR-T cells from infusion products and PB	scRNA-seq scTCR-seq	10 × Genomics Illumina Hiseq 2500	Clonal kinetics and transcriptional programs regulate the fate of CAR-T cell after infusion	[186]
B-ALL	BM	scRNA-seq	10 × Genomics Illumina NextSeq 500	A Darwinian-like selection of preexisting CD19^neg^ B-ALL cells is a mechanism for CD19neg B-ALL relapse after CAR-T cell therapy	[189]
LBCL	Pre-treatment and post-treatment PB samples	scRNA-seq scTCR-seq CITE-seq	10 × Genomics Illumina NovaSeq 6000 or HiSeq 4000	Helios-expressing population of circulating CD4^+^ CAR-T cells on day 7 is associated with clinical progression and reduced neurotoxicity, and this population is non-clonal and manifests hallmark features of T regulatory cells. CAR-Treg cells and tumor burden surrogate can identify patients with clinical progression	[40]
B-ALL	Infusion products	scRNA-seq CITE-seq	DropSeq Illumina HiSeq 4000	A deficiency of T helper 2 function was associated with CD19-positive relapse compared with durable responders The frequency of early memory T cells, rather than activation or co-inhibitory signatures, could distinguish the relapse These findings were corroborated by independent functional profiling of 49 patients, and an integrative model was developed to predict the response	[225]
NHL	CAR-T cells pre- and postinfusion	scRNA-seq combined with TotalSeq™-B antibodies	10 × Genomics	The evolution of CAR-T cells was toward a non-proliferative, highly differentiated, and exhausted state. An enriched exhaustion profile in CAR-T cells of patients with poor response was marked by TIGIT expression	[158]
MM	FACS sorted CAR-T cells from PB samples post treatment	scATAC-seq	10 × Genomics Illumina NovaSeq 6000	BATF and IRF4 are pivotal regulators in CAR-T cell exhaustion and reducing the expression of BATF or IRF4 had benefits to improve antitumor potency of CAR-T cells	[33]
LBCL	Infusion products (axi-cel)	scRNA-seq scTCR-seq	10 × Genomics Illumina HiSeq4000	The heterogeneity in the cellular and molecular features of CAR-T cell infusion products contributes to variation in efficacy and toxicity after axi-cel therapy in LBCL, and that day 7 molecular response might serve as an early predictor of CAR-T cell efficacy A rare cell population with monocyte-like transcriptional features was associated with high-grade ICANS	[41]
B cell malignancies	Human brain, lung and PBMC, mouse whole dissociated brain	scRNA-seq	Public data from GEO 10 × Genomics Illumina HiSeq 2500	CD19, primarily considered as a B cell-specific surface antigen, is expressed in human brain mural cells that are critical for blood–brain-barrier integrity, suggesting that this cell population may contribute to the neurotoxicity of CD19-directed immunotherapy including CAR-T	[125]
Richter-transformed DLBCL	Pre-treatment and post-treatment PB samples	scRNA-seq scTCR-seq	10 × Genomics Illumina NovaSeq	Highlight the complex nature of CAR-T-related hematological toxicity and introduce oligoclonal CAR-T cell expansion as a potential contributing pathophysiologic mechanism	[226]
B cell lymphoma	Infusion products	scRNA-seq	Public data from GSE150992	Neurotoxicity is associated with decreasing cycling activity, amount of CAR + cells, and expression of cell cycle genes and exhaustion related genes	[227]

*GEO* Gene Expression Omnibus, *HCL* Human Cell Landscape, *AHCA* adult human cell atlas, *AML* acute myeloid leukemia, *CSF1R* colony-stimulating factor 1 receptor, *CD86* cluster of differentiation 86, *PBMC* peripheral blood mononuclear cell, *CITE-seq* cellular indexing of transcriptomes and epitopes by sequencing, *scFTD-seq* single-cell freeze–thaw lysis directly toward 3′ mRNA sequencing, *scATAC-seq* single-cell Assay for Transposase-Accessible Chromatin using sequencing, *TME* tumor microenvironment, *CyTOF* mass cytometry by time-of-flight, *PB* peripheral blood*, BM* blood marrow, *B-ALL* B-cell acute lymphoblastic leukemia, *NHL* non-Hodgkin lymphoma, *FACS* fluorescence activating cell sorter, *axi-cel* axicabtagen ciloleucel, *LBCL* large B-cell lymphoma, *ICANS* Immune effector cell-associated neurotoxicity syndrome, *TCF7* transcription factor 7, "-" not available

## Single-cell analysis of CAR-T cell therapy at the preclinical stage

**Fig. 1:**
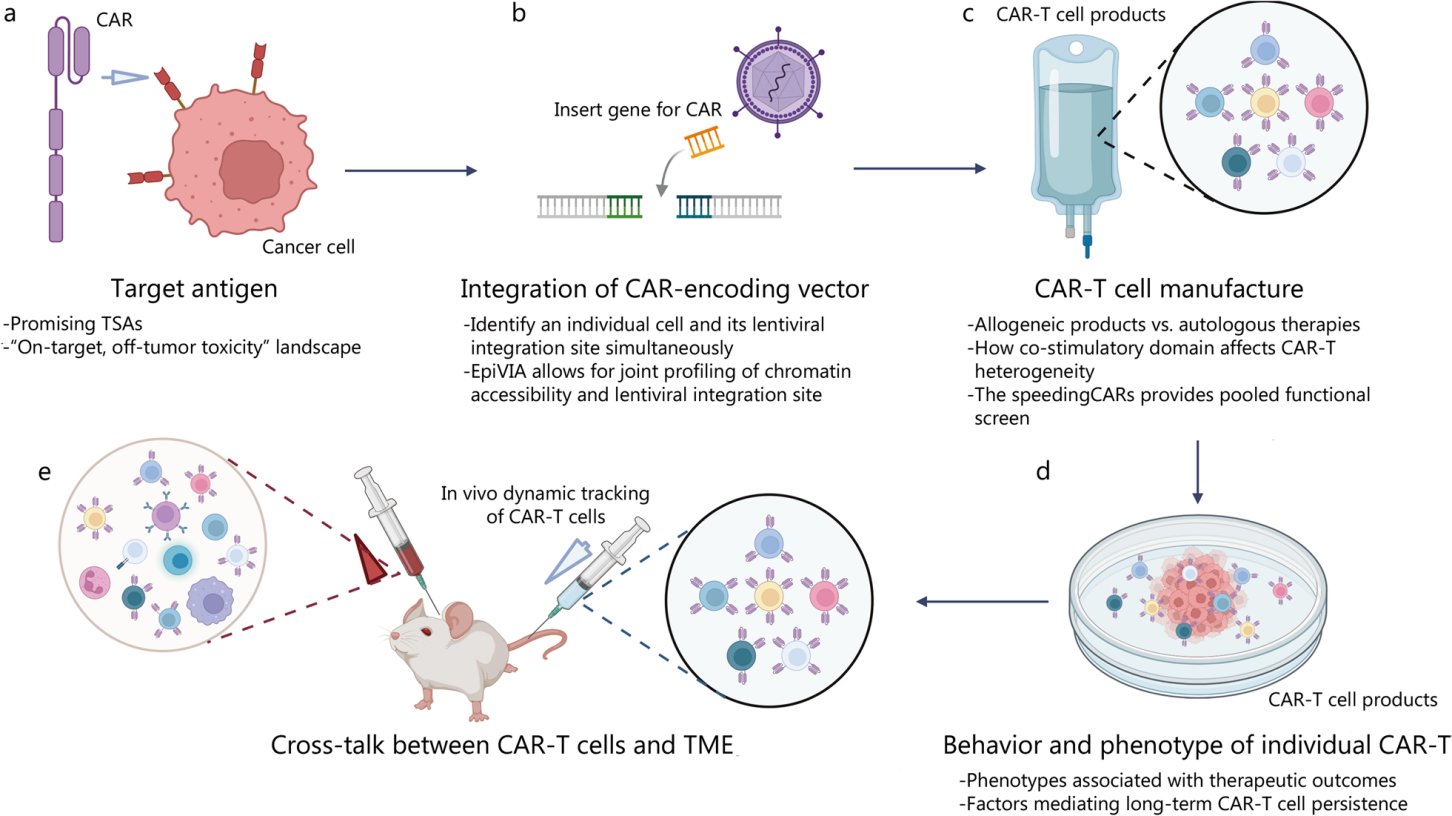
Outline of single-cell sequencing applied to interrogate CAR-T cell biology in preclinical studies. Single-cell sequencing has been applied to interrogate CAR-T cell biology with in vitro and in vivo preclinical studies. **a** To discover promising TSAs; **b** To chart where the CAR-T vector integrates into the genome; **c** To unveil heterogeneities in the transcriptional, phenotypic, functional, and metabolic profiles of CAR-T cells; **d** Single-cell profiling of the behavior and phenotype of pre-infusion CAR-T cells; **e** Studying CAR-T cells in vivo and the cross-talk between CAR-T cells and TME. CAR chimeric antigen receptor, TSA tumor-specific antigens, TME tumor microenvironment

The discovery of CAR-T cells in the preclinical stage was based on in vitro co-culture assays and in vivo xenograft mouse models. Although these conventional assays could be used to evaluate the expression of cell surface markers, cytokine secretion, and cancer cell killing, the spectrum of individual CAR-T cell behaviors cannot be fully covered. Single-cell signatures from tumor biopsies, T cell apheresis products, and infusion products can be used to determine the predictive features of clinical responses before therapy and guide personalized therapies. Can single-cell sequencing be established as a tool to explore ideal target antigens, evaluate new CAR designs, and guide personalized treatment? In the next section, we will discuss the application of single-cell sequencing to explore the biology of CAR-T cells through in vitro and in vivo preclinical studies (Fig. 1).

### Target antigens of CAR-T cells

CAR-T immunotherapy has the advantage of higher targeting specificity than conventional chemotherapy and radiotherapy. An increasing number of CAR-T cell products are being developed for the treatment of cancer. Despite their universal utility, the target antigens are also expressed in normal cells, such as CD19 in the normal B-cell lineage. The "on-target, off-tumor" toxicity of CAR-T therapy has been widely reported, although a majority of others have not been identified or are overlapped with other symptoms [216, 228]. An understanding of the consequences of "on-target, off-tumor" toxicity is also essential for the development of a safe and effective therapy [86, 229]. Some "on-target, off-tumor" toxicities were not anticipated in preclinical animal studies because of the variability in cross-species reactivity to nonhuman target antigens. This usually leads to a false report of safety. In summary, it is essential to design CAR-T cell products that target cancer cells with negligible off-tumor toxicity.

Single-cell sequencing is a potent tool for studying various cell subsets, investigating rare cell types, and dissecting complex regulatory networks and developmental trajectories. Comprehensive single-cell multiomics studies with both tumor and normal tissues are helping us to gain new insights for predicting toxicities and guiding the medical practice of CAR-T therapy [169, 221, 230]. Jing et al. [217] designed a user-friendly data portal, CAR-target gene toxicity at the single-cell level (CARTSC), to browse and search for single target and examine the expression patterns of selected target genes in immune cells and tissues. This study was based on single-cell analysis and presented a potential "on-target, off-tumor" toxicity landscape for CAR targets in a wide range of tissues (Fig. 1a). It was recommended that these targets should be considered in advance of CAR design to avoid "on-target, off-tumor" toxicity and that symptoms should be closely monitored to identify early signs of toxicity. Zhang et al. [216] analyzed data from 18 normal tissues and organs and CAR-targeted antigen expression patterns using publicly available scRNA-seq datasets to determine which normal cells might be inappropriately targeted by CAR-T cells. This comprehensive single-cell atlas characterizes target antigens expressed on some normal cell types, helps us capture antigen-expressing rare cell types, and facilitates elucidation of the underlying cause of "on-target, off-tumor" toxicity in specific tissues and organs. In another study, the researchers leveraged an atlas of publicly available RNA sequencing data of over 500,000 single cells from 15 individuals with AML and tissue from 9 healthy individuals and identified CSF1R (colony-stimulating factor 1 receptor) and CD86 (cluster of differentiation 86) as targets for CAR-T cell therapy in AML [221]. Extensive in vitro and in vivo validation assays demonstrated their widespread expression on AML blasts, the powerful and long-lasting treatment responses of newly developed CAR-T cells in vitro and in vivo, and their minimal toxicity to relevant healthy cells and tissues [221]. As a result, predictions can be made about the "on-target, off-tumor" toxicity of CAR-T therapies, providing guidance on preventive measures to be taken during CAR-T treatment.

### Integration of CAR-encoding vectors

The therapeutic failures reported in most clinical trials were due to limited expansion of CAR-T cells. The site of lentiviral vector integration has been reported to affect clonal expansion, and the site of vector integration within the *TET2* gene has been shown to be the key contributor to unique clonal expansion in an individual patient [62]. Therefore, the precise localization of the CAR vector in the genome of the T cell is a critical determinant of the outcome of the treatment [63, 231]. Two methods are widely used for lentiviral integration site analysis: ligation-mediated and linear amplification-mediated PCR, which involves ligation of a linker DNA cassette to fragmented genomic DNA and enables PCR amplification between known sequences in a long terminal repeat and linker DNA in viruses [232, 233]. However, these techniques, somewhat disappointingly, cannot simultaneously characterize the identity of an individual cell and its lentiviral integration site [231–235]. To investigate whether CAR integration enhances the proliferation of individual T cells, it is critical to define the identity of an individual CAR-T cell while also determining where the CAR-T vector is being integrated into the genome (Fig. 1b).

Wang et al. developed a method, called EpiVIA, for joint profiling of chromatin accessibility and lentiviral integration site identification at the cell population and single-cell levels, which enabled the discovery of cellular fates associated with durable CAR-T responses (Fig. 1b). This technique was also validated in clonal cells with predefined integration sites. In addition, the technique was used to determine the number of lentiviral integration sites and host chromatin accessibility at single-cell resolution in CAR-T cells [222]. The scRNA-seq is also helping to optimize the integration of CAR-encoding vectors. A "two-in-one" approach has been developed to generate nonviral, gene-specific, targeted CAR-T cells using CRISPR/Cas9 technology [218]. The scRNA-seq was performed in CAR-T cells to characterize nonviral, PD-1-integrated CAR-T cells and revealed that the electroporation method resulted in a high percentage of memory T cells among the infused products and that PD-1 interference enhanced antitumor immune functions [218]. Charitidis's team developed a scRNA-seq approach to monitor the transfer of a CD19 CAR gene by lentiviral vectors, i.e., the conventional vesicular stomatitis virus lentiviral vector and the CD8-targeted lentiviral vector [34]. The data indicated trimodal expression for the CAR and CD8A and the upregulation of restriction factors in CAR-negative cells, thereby accounting for their protection from CAR gene transfer. These findings shown that scRNA-seq provides a workflow and subset-identifying approach to reliably distinguish transduced from untransduced cells during CAR-T cell manufacturing.

### CAR-T cell manufacturing

It remains to be determined how the intrinsic heterogeneities of the engineered cells regulate the therapeutic efficacy and whether allogeneic products can match the efficacy of autologous therapies. Bai's team used scRNA-seq in combination with CITE-seq to reveal heterogeneities in the transcriptional, phenotypic, functional and metabolic profiles of donor and patient CAR-T cells at baseline or after antigen-specific activation (Fig. 1c) [35]. After CD19 stimulation, donor CAR-T cells showed a more pronounced activation level in correlation with the upregulation of major histocompatibility complex class II genes compared with patient CAR-T cells [35]. This integrated multiomics profiling provides a mechanistic basis for the development of next-generation "off-the-shelf" allogeneic products.

Furthermore, the differentiation state and costimulatory domain during manufacturing may also affect the CAR-T cell function. Xhangolli et al. [236] employed high-throughput single-cell transcriptome sequencing, multiplexed single-cell cytokine secretion assays, and live cell imaging of cytolytic activity, to study CAR-T cells upon antigen-specific stimulation. Both CD4^+^ and CD8^+^ CAR-T cells were equally capable of exerting cell-mediated cytotoxicity regardless of the differentiation state, and the activation of CAR-engineered T cells was found to be a canonical process that led to a highly mixed response involving both type 1 and type 2 cytokines together with granulocyte–macrophage colony stimulating factor. CAR tonic signaling, from CD28z and 4-1BBz CARs, could skew the predominance of particular T cell subsets within resting CAR-T cells, and 4-1BBz CARs were enriched in CD8^+^ central memory cells in contrast to CD8^+^ effector and CD4^+^ central memory cells in the CD28z CAR^+^ T cell population [219]. These results indicate that a costimulatory domain may influence the heterogeneity of unique T cell transcriptional profiles. Castellanos-Rueda's team generated a library of 180 unique CAR variants that were genomically integrated into primary human T cells using CRISPR-Cas9 (Fig. 1c) [220]. The speedingCARs library provides an integrated approach to CAR-T cell engineering via signaling domain shuffling and pooled functional screening [220]. scRNA-seq and single-cell CAR sequencing (scCAR-seq) provide high-throughput screening to discover multiple variants with tumor-killing properties and T cell phenotypes that are significantly different from standard CARs, helping to expand the combination space of CAR signaling domains and supporting the speedingCARs as a tool for the engineering of CARs for potential therapeutic development [220].

### Behaviors and phenotypes of individual CAR-T cells

Cellular heterogeneity in infusion products plays a critical role in the varying efficacy of CAR-T cell therapy. Understanding the killing behavior and phenotype of individual CAR-T cells has been a major challenge. LaBelle et al. [237] have developed a platform to measure time-dependent CAR-T cell-mediated cytotoxicity and then isolate single cells for downstream assays, which would be extremely valuable in the characterization of CAR-T cells (Fig. 1d). Given that single-cell resolution accurately captures the degree of similarity between samples, it will be of great importance to study cellular heterogeneity and adequately characterize the interplay among T cell subsets in CAR-T cell therapy. Xue et al. [223] used a single-cell barcode chip microdevice to demonstrate the diverse landscape of the immune response of CAR-T cells, providing a new platform for capturing CAR-T product data for correlative analysis. A comprehensive evaluation of pre-infusion products paves the way for understanding the relationship between in vitro functional profiles and therapeutic outcomes.

More recently, Wang’s team performed scRNA-seq to explore the T cell phenotypes associated with different stages of the production of dual BCMA (B cell maturation antigen)- and TACI-targeting CAR-T cells [238]. This study demonstrated that tonic signaling occurs in a small proportion of unactivated CAR-T cells and identified persistent and distinctive CAR-induced molecular signatures of T cell activation characterized by high MYC transcription factor-induced gene expression, limited exhaustion, and a combination with CD4^+^ CD8^+^ effector response. Qin et al*.* [239] designed a kind of CAR-T cells co-expressing chimeric switch receptors specific for PD-L1 and revealed that these CAR-T cells could promote differentiation into central memory-like T cells, upregulate genes related to T helper 1 (Th1) cells and downregulate Th2-associated cytokines through the CD70-CD27 axis. More importantly, integrative bulk and single-cell profiling of premanufactured T cell populations revealed that some factors mediated the long-term persistence of CAR-T cells (Fig. 1d). The researchers performed RNA-sequencing analysis on sorted T cell subsets from all 71 patients, followed by paired CITE-seq and scATAC-seq on T cells from six of these patients. The chronic interferon signaling regulated by interferon regulator factor 7 (IRF7) was related to poor CAR-T cell persistence across T cell subsets, and the transcription factor 7 (TCF7) regulon was not only associated with the favorable naïve T cell state but also with a high number of effector T cells maintained in patients with long-term CAR-T cell persistence [239]. Altogether, single-cell profiling of the behaviors and phenotypes of pre-infusion CAR-T cells may provide critical insights into the underlying molecular determinants and predictors of treatment outcomes.

### Cross-talk between CAR-T cells and TME

The TME is a contributor to cancer progression and relapse and is also strongly associated with the failure of cancer immunotherapy [240–244]. CAR-T cells require interactions with the TME but do not act autonomously. To promote endogenous immune responses, cellular cross-talk between CAR-T cells and the host may be essential (Fig. 1e) [194, 245, 246]. Traditional CAR-T cell studies use in vitro and in vivo experiments to assess cell surface marker expression, cytokine secretion, and tumor cell killing. However, these assays do not fully reflect the effect of T cell immune surveillance and its interactions with the TME [243, 247]. Single-cell sequencing technologies have served as a powerful tool for studying CAR-T cells in vivo due to their high resolution and unbiased detection. Boulch et al. [194] used intravital microscopy to visualize in situ interactions between tumors and anti-CD19 CAR-T cells. In an immunocompetent mouse model of B-cell lymphoma, single-cell RNA sequencing was used to examine subsequent changes in the TME, revealing significant changes in the TME during CAR-T cell therapy [194]. While CD4^+^ CAR-T cells were more effective in stimulating the host immune response, CD8^+^ CAR-T cells excelled in direct tumor cell killing, both of which required CAR-T cell-intrinsic expression of IFN-γ (interferon-γ). Host sensing of IFN-γ and production of IL-12 were also required for CAR-T cell function, providing further support that cross-talk between CAR-T cells and the TME is necessary for optimal CAR-T cell efficacy against tumors [194]. Therefore, an attractive strategy to prevent relapse after therapy is to enhance the interaction of CAR-T cells with the host.

## CAR-T cell behaviors throughout clinical treatment correlate with therapeutic response

**Fig. 2:**
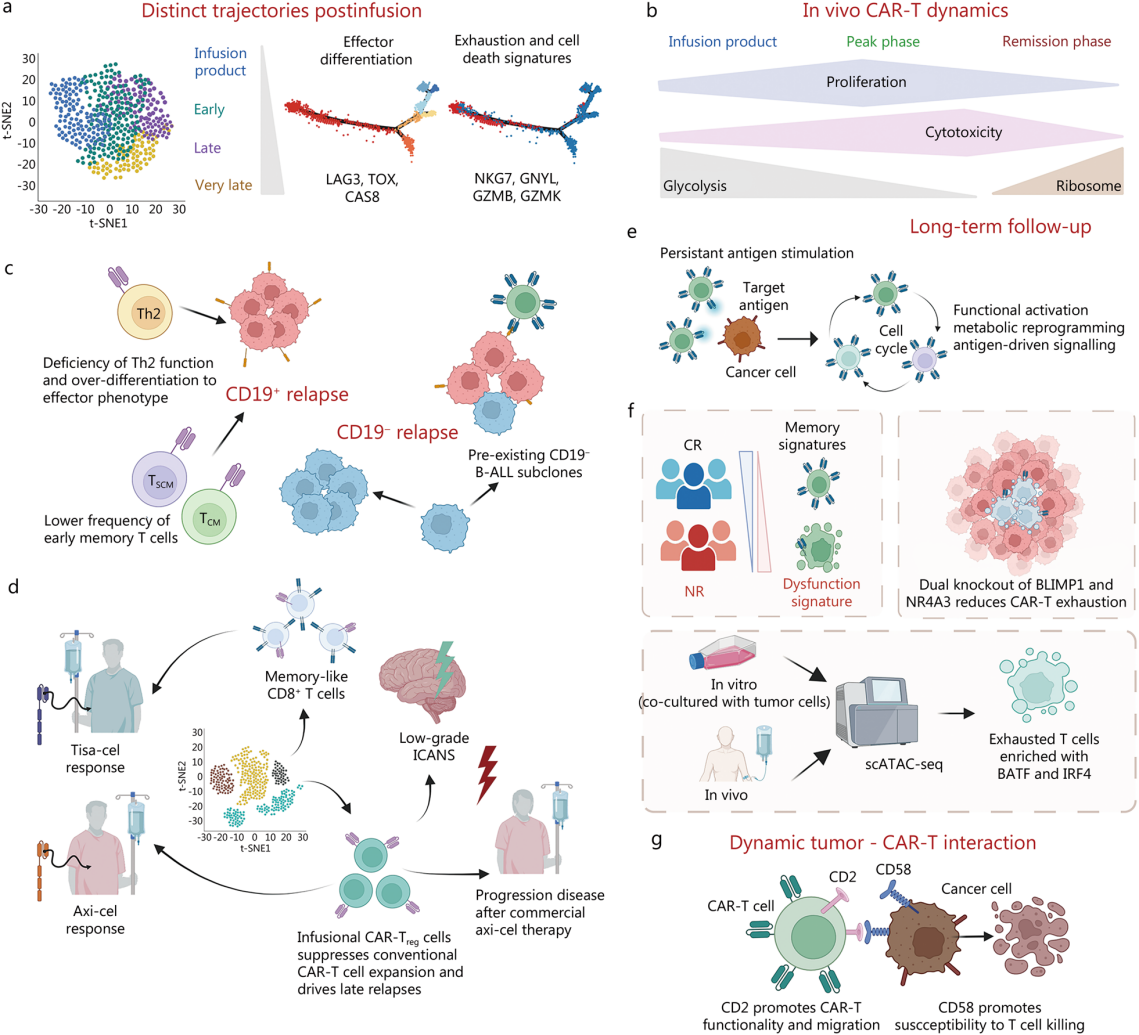
CAR-T cell behaviors throughout clinical treatment correlate with therapeutic response. Single-cell sequencing has been applied to interrogate in vivo clonal kinetics and molecular profiles that regulate the fate of CAR-T cells after infusion. **a** Different CAR-T cell subsets lead to divergent differentiation trajectories after infusion [186, 187]; **b** A model of in vivo dynamic changes of CAR-T cells post infusion was proposed [192]; **c** The phenotypes of CAR-T cells were associated with CD19-positive relapse [225] and CD19-negative relapse [189]; **d** Transcriptional mechanisms of response vary by CAR-T products [224]; **e** The long-persisting CD4^+^ CAR-T cells exhibited cytotoxic characteristics along with proliferation, cytokine expression, metabolic activity, and in vitro response to CAR stimulation [191]; **f** CAR-T cell exhaustion during exposure to prolonged tumor antigen was considered as one of the major causes resulting in tumor progression and immunotherapy failures [41]; **g** CD2 on T cells was associated with directional migration, and the interaction between CD2 on T cells and CD58 on lymphoma cells accelerated serial killing. LAG3 lymphocyte activation gene-3, TOX thymocyte selection-associated high mobility group box, CAS8 caspase 8, NKG7 natural killer cell protein 7, GNYL Granulysin, GZMB Granzyme B, GZMK Granzyme K, CAR-T chimeric antigen receptor T, Th2 T helper 2, T_SCM_ T memory stem cell, T_CM_ central memory T cell, B-ALL B-cell acute lymphoblastic leukemia, tisa-cel tisagenlecleucel, axi-cel axicabtagene ciloleucel, ICANS immune effector cell-associated neurotoxicity syndrome, T_reg_ regulatory T cell, CR complete response, scATAC-seq single-cell assay for transposase-accessible chromatin using sequencing, NR no response, BLIMP1 B lymphocyte-induced maturation protein 1, NR4A3 nuclear receptor subfamily 4 group A member 3, BATF basic leucine zipper ATF-like transcription factor, IRF4 interferon regulatory factor 4

With promising effects achieved in preclinical studies, a great many ongoing clinical trials have demonstrated their efficacy and safety profiles. CAR-T cells are exposed to a dynamic TME and are consistently triggered by antigen-expressing cancer cells after infusion. The in vivo persistence and dynamics of transferred CAR-T cells is predictive of long-lasting antitumor immunity and therapeutic outcomes. However, clonal kinetics and molecular profiles of infused T cells that regulate the fate of CAR-T cells after infusion remain poorly understood. Moreover, how the host and the changing tumor burden affect the state of CAR-T cells over time is also not fully elucidated. High-resolution views of gene expression, cell heterogeneity, development trajectory, and cell lineage tracing may help to address these issues. The loss of T cell stemness, poor expansion capacity, and cell exhaustion during prolonged exposure to tumor antigens are major causes of therapeutic resistance. Understanding the predictive molecular profiles associated with a therapeutic response will help us gain insights for optimizing CAR design and manufacturing and identify patients who will benefit more from the CAR-T therapy (Fig. 2).

### Dynamic tracking of CAR-T cells in vivo

How CAR-T cell phenotypes vary from the pre-infusion product throughout the course of treatment requires further investigation. Single-cell sequencing has the potential to offer unique insights into the in vivo behavior and dynamics of CAR-T cells after adoptive cell transfusion [248]. Using scRNA-seq in combination with TCR repertoire sequencing, the researchers were able to track the fate of individual CAR-T cell clonotypes between infusion products and blood over time after infusion. Using the endogenous TCR as a lineage barcode for CAR-T cells, the researchers discovered that infused CAR-T cells were distinct from CAR-T cells in the blood [186, 187]. Clones that expand and persist in the blood beyond 3 months after infusion are mainly derived from infused clusters with a higher expression of cytotoxicity and proliferation-related genes [186, 187]. A pseudotime analysis revealed two distinct trajectories of CAR-T cell differentiation after infusion: one trajectory involves effector differentiation characterized by the expression of conventional cytotoxic genes; the other trajectory indicates the rapid development of the same exhaustion and cell death signatures soon after infusion (Fig. 2a) [186, 187]. Both anti-BCMA CAR-T and anti-CD19 CAR-T had similar transcriptional characteristics [249]. A model for the dynamic in vivo changes of CAR-T cells after infusion was proposed (Fig. 2b) [192]. Initially, the infusion products were highly metabolically active, with high glycolysis and biosynthetic gene expression. CAR-T cells in the peak phase gradually transitioned from a highly proliferative state to a highly cytotoxic state along a developmental trajectory. In the late remission phase, CAR-T cells were not proliferative but maintained their cytotoxicity. Eventually, the proliferation and cytotoxicity signatures in the CAR-T cells declined in the remission phase [192]. Notably, most studies have focused on a limited number of phenotypic markers in blood and infusion products, which provide an incomplete view of the complexity of CAR-T cells. Goldberg et al. adapted the mass cytometry to simultaneously analyze trafficking and functional protein expression in CD19 CAR-T cells in a wide range of patient tissues, including leukapheresis T cells, CAR product, CAR-T cells in peripheral blood, bone marrow, and cerebrospinal fluid after infusion, and they correlated them with immune phenotypes. With leukapheresis T cells as a baseline, patients’ infusion products showed an upregulation in many trafficking and activation molecules. Furthermore, cerebrospinal fluid samples were significantly enriched in CD4 and CD8 trafficking and memory phenotype proteins when compared to peripheral blood samples. These findings revealed the spatiotemporal plasticity of CAR-T cells and provided a potential framework for CAR-T cell remodeling and enhanced immunotherapy efficacy [203].

### Response-associated CAR-T cell behaviors

Within one year after CAR-T cell therapy, a notable number of ALL (acute lymphoblastic leukemia) patients develop CD19-positive relapse. Bai et al. described single cell transcriptomes and surface protein landscapes of infusion products from 12 ALL patients and observed substantial heterogeneity in antigen-specific activation states, among which deficient Th2 cell function and excessive differentiation into effector cell phenotypes were associated with CD19-positive relapse (Fig. 2c). These results may provide some clues for the approach to prolong the response duration of CAR-T therapy. The proteomic data also revealed that a lower frequency of early memory T cells could predict relapse [225]. Approximately half of relapsed patients develop CD19-negative B-ALL, allowing leukemic cells to evade CD19-targeted CAR-T therapy. Rabilloud's team reported the scRNA-seq data obtained from an analysis of leukemic samples from a B-ALL patient before and after CAR-T therapy (Fig. 2c). The presence of pre-existing CD19-negative B-ALL subclones prior to CAR-T treatment, which may provide new ways to assess the risk of failure of targeted therapy [189].

Haradhvala’s team performed scRNA-seq and scTCR-seq of 105 samples from 32 LBCL (large B-cell lymphoma) patients treated with axi-cel (axiabtagene ciloleucel) or tisa-cel (tisagenlecleucel), and these samples were collected throughout the treatment (Fig. 2d) [224]. The two products had different designs and manufacturing processes (4-1BB vs. CD28 co-stimulation, CD8 vs. CD28 transmembrane for tisa-cel vs. axi-cel) and were produced by different vectors and manufacturing processes (fresh vs. frozen apheresis products, activation by antibody-coated beads vs. soluble antibodies and cytokines). This study revealed that the cellular dynamics of the responses differed significantly between the two products: expansion of proliferative memory-like CD8 clones was a hallmark of the tisa-cel response, whereas axi-cel responders exhibited more heterogeneous populations [224]. Increased frequencies of CAR-T regulatory (CAR-Treg) cell populations were observed in axi-cel non-responders, and these subpopulations were also able to suppress conventional CAR-T cell expansion and drive late relapses in an in vivo model [40, 224]. In addition, by performing single-cell proteomic profiling of circulating CAR-T cells from 32 LBCL patients receiving commercial axi-cel, it was found that higher levels of CD4^+^ Helios^+^ CAR-T cells at day 7 after infusion correlated with clinical progression and milder neurotoxicity [40]. Taken together, these findings elucidate the single-cell resolution, and response-associated characteristics of each CAR-T cell product, allowing us to optimize CAR-T design and match patients to the treatments most likely to induce better clinical outcomes.

### CAR-T cell exhaustion and persistence

T cells can be categorized in terms of the stage of cell differentiation, which is correlated with therapeutic efficacy and their in vivo persistence [250]. Long-lasting CD19-redirected CAR-T cells were detectable more than ten years after infusion in two CLL patients who achieved sustained remission (Fig. 2e) [191]. Single-cell profiling indicated that these long-lasting CD4^+^ CAR-T cells exhibited cytotoxic characteristics along with proliferation, cytokine expression, metabolic activity, and in vitro response to CAR stimulation, which suggested that these long-lasting CAR-T cells remained functionally active rather than exhausted [191]. The apparent cloning selection could be due to the silencing of neighboring genes, integration in regions of the genome linked to more robust expression of the CAR construct and genetic drift.

T cell exhaustion during prolonged exposure to tumor antigens is considered one of the major causes of tumor progression and immunotherapy failures. Exhaustion-related transcription factors act as central regulators that affect the expression of immune checkpoint genes and drive T cell exhaustion [251–256]. A growing number of transcription factors have been found to be implicated in the process of chronic viral infection or tumor burden-induced T cell exhaustion [252–257]. The scATAC-seq analysis from serial tumor biopsies before and after PD-1 blockade identified chromatin regulators of therapy-responsive T cells and revealed a shared regulatory program that governed intra-tumoral CD8^+^ T cell exhaustion and CD4^+^ T follicular helper cell development [172]. However, the process of T cell exhaustion differs considerably between CAR-T cells and non-transformed T cells. Therefore, the transcription factors that play an important role in the exhaustion of non-transformed T cells should be further verified in CAR-T cells.

The scRNA-seq data on infusion products from LBCL patients treated with axi-cel demonstrated that complete clinical response was correlated with higher frequencies of CD8^+^ T cells expressing memory signatures, whereas poor clinical response was associated with a CD8^+^ T cell dysfunction signature enriched for exhaustion and activation markers and genes encoding MHC class II proteins (Fig. 2f) [41]. Markers on tumor biopsy samples can also be used to predict the dysfunction of CAR-T cells in response to the TME. Singh et al. demonstrated that treatment resistance is associated with the upregulation of exhaustion markers [57]. Furthermore, the scRNA-seq of CAR-T cells from a first-in-human trial in metastatic prostate cancer identified two independently validated cell states associated with antitumor potency (Fig. 2f) [188]. The evolution of CAR-T cells was towards a non-proliferative, highly differentiated and exhausted state. An enriched exhausted profile in CAR-T cells from patients with unsatisfactory response was characterized by TIGIT expression [158]. Two transcription factors, BLIMP1 (B lymphocyte-induced maturation protein 1) and NR4A3 (nuclear receptor subfamily 4 group A member 3), may regulate CAR-T cell dysfunction. Dual knockout of the two transcription factors, which outperformed single knockout alone, shifted CAR-T cell phenotypes away from TIM-3^+^ CD8^+^ and toward TCF1^+^ CD8^+^ to counteract exhaustion of tumor-infiltrating CAR-T cells, and enhanced their antitumor activity in multiple mouse models [188]. With initially observed CAR-T cell differentiation and increased exhaustion after co-culture with tumor cells in vitro, Jiang et al. then performed scATAC-seq to comprehensively and dynamically depict the landscape of chromatin accessibility of CAR-T cells during tumor cell stimulation. BATF (basic leucine zipper ATF-like transcription factor) and IRF4 (interferon regulatory factor 4) were significantly enriched in terminally exhausted CAR-T cells both in vitro and in vivo, suggesting that disruption of BATF or IRF4 expression may inhibit CAR-T cell differentiation to the extent of terminal exhaustion (Fig. 2f) [33]. Therefore, to achieve better therapeutic outcomes, exhaustion-related transcription factors in CAR-T cells may be epigenetically modified to mitigate exhaustion and prolong in vivo persistence.

### Interactions between CAR-T cells and tumor cells

Romain et al. [258] investigated the importance of dynamic interactions between T cells and tumor cells in facilitating clinically relevant discoveries with high-throughput single-cell technologies based on time-lapse imaging microscopy in nanowell grids (TIMING), integrating the killing, cytokine secretion and transcriptional profiling assays. Directional migration of CD19-specific CAR-T cells was found to be correlated with multifunctionality (Fig. 2g) [258]. Their results demonstrated that CD2 on T cells was related to the directional migration of T cells. The interaction between CD2 on T cells and CD58 on lymphoma cells accelerated serial killing, highlighting the necessity of studying dynamic CAR-T-tumor cell interactions [258].

## Underlying mechanisms of toxicities based on single-cell analyses

**Fig. 3:**
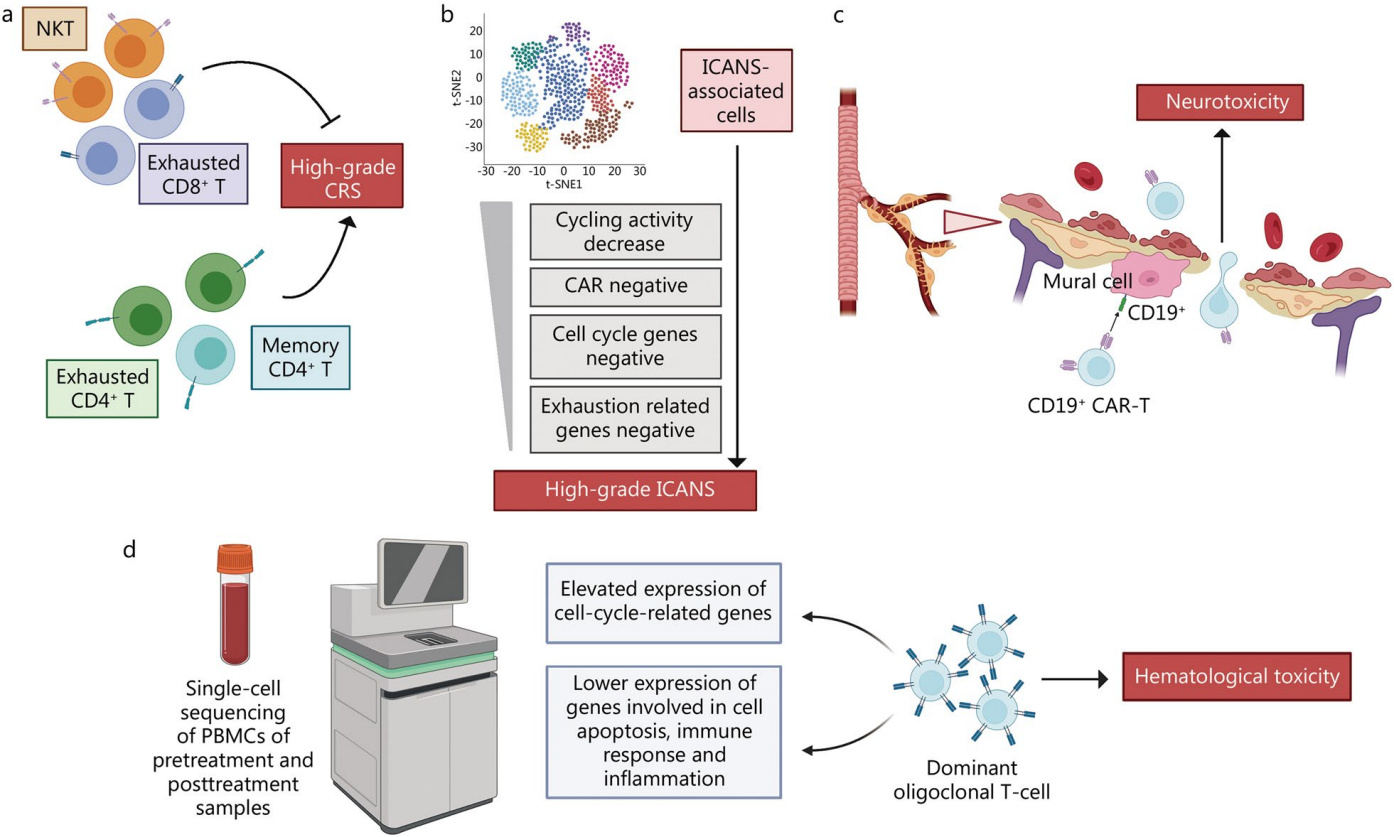
Underlying mechanisms of toxicities based on single-cell analyses. **a** High-grade CRS is associated with reduced frequencies of exhausted CD8^+^ T cells and NKT cells and increased frequencies of exhausted and memory CD4^+^ T cells [41]; **b** ICANS-associated cells were significantly over-represented in the infusion products of patients who developed high-grade ICANS [125]; **c** CD19^+^ human brain mural cells may contribute to the neurotoxicity of CD19-directed CAR-T immunotherapy; **d** Hematological toxicity is associated with the dominant oligoclonal T cell expansion [226]. CRS cytokine release syndrome, NKT natural killer T, ICANS immune effector cell-associated neurotoxicity syndrome, CAR-T chimeric antigen receptor T, PBMCs peripheral blood mononuclear cells

With the increasing clinical efficacy of the therapies, the immune responses induced by the immunotherapies are also causing a series of adverse effects, in particular the CRS and ICANS that are often associated with CAR-T cell therapies [89, 91, 93, 94, 259–261]. As noted above, multi-organ failure associated with CRS can be fatal in some cases. Although it is reversible in the majority of cases, neurotoxicity prolongs the hospital stay, requires intensive care in a subset of patients, delays the recovery, and increases the cost of care [41]. Combined with clinical parameterization, single-cell sequencing allows a more comprehensive characterization of cellular subpopulations and transcriptomic states and thus greater understanding of their relationship to adverse effects (Fig. 3).

Deng et al. [41] identified characteristics of the CAR-T cell infusion products that were substantially correlated with toxicities. Although the proportion of their patients with high-grade CRS was small, they observed a negative association with exhausted CD8 T cells and a positive association with exhausted CD4^+^ T cells (Fig. 3a) [41]. Patients with high-grade ICANS presented with a significantly reduced frequency of CAR^+^ cells within their infusion products. They also identified a rare monocyte-like cell population within axi-cel infusion products that were significantly correlated with the development of high-grade ICANS [41]. These cells herein are referred to as ICANS-associated cells (Fig. 3b), and this novel mechanism by which discrete populations of cells give rise to high-grade ICANS may pave the way for therapeutic intervention to reduce toxicity following CAR-T cell infusion.

One study from the Stanford University School of Medicine used single-cell analysis to identify brain mural cells expressing CD19, which surround the endothelium and are critical for blood–brain barrier integrity (Fig. 3c) [125]. CD19 expression in the brain begins early in development with the emergence of mural cell lineages. It persists throughout adulthood in all brain regions. However, a limitation of preclinical animal models of neurotoxicity is based on mouse mural cells expressing lower levels of CD19. These findings highlight the utility of human single-cell atlases for the design of immunotherapies and suggest an "on-target" mechanism underlying neurotoxicity in CD19-targeted therapies. Re-analysis of scRNA-seq data from 24 patients focused on cellular states and their association with immune cell-related neurotoxicity [227]. Neurotoxicity was associated with a decreasing cell cycling activity, amount of CAR + cells, and expression of cell cycle and exhaustion-related genes *LAG3* and *TIM3*, providing molecular details of the transcriptomic landscape that may be targets for overcoming neurotoxicity [227].

Likewise, the real-world evidence has also highlighted the incidence of hematological toxicity induced by CAR-T therapy [226, 248]. Cytopenia usually occurs long after lymphodepleting chemotherapy and the resolution of CRS and is often prolonged and biphasic. Worse, severe bone marrow failure can predispose to serious infections and high non-relapse mortality. A 57-year-old patient with Richter's transformation DLBCL (diffuse large B-cell lymphoma) received tisa-cel as standard of care. Using combined scRNA-seq and scTCR-seq, their report highlighted the complex nature of CAR-T-related hematologic toxicity and proposed oligoclonal CAR-T cell expansion as a potentially contributing pathophysiologic mechanism [226]. Hematologic toxicity is associated with dominant oligoclonal T cell expansion in both CAR- and non-CAR-bearing T cell populations, as demonstrated by elevated expression of cell cycle-related genes and decreased expression of genes involved in cell apoptosis, immune response, and inflammation (Fig. 3d) [226]. In addition, monocyte loss was also found in two patients who died from severe infections, indicating that monocyte loss may be a primary cause of infections after CAR-T infusion [248].

## How will single-cell sequencing affect the future of CAR-T therapy?

**Fig. 4:**
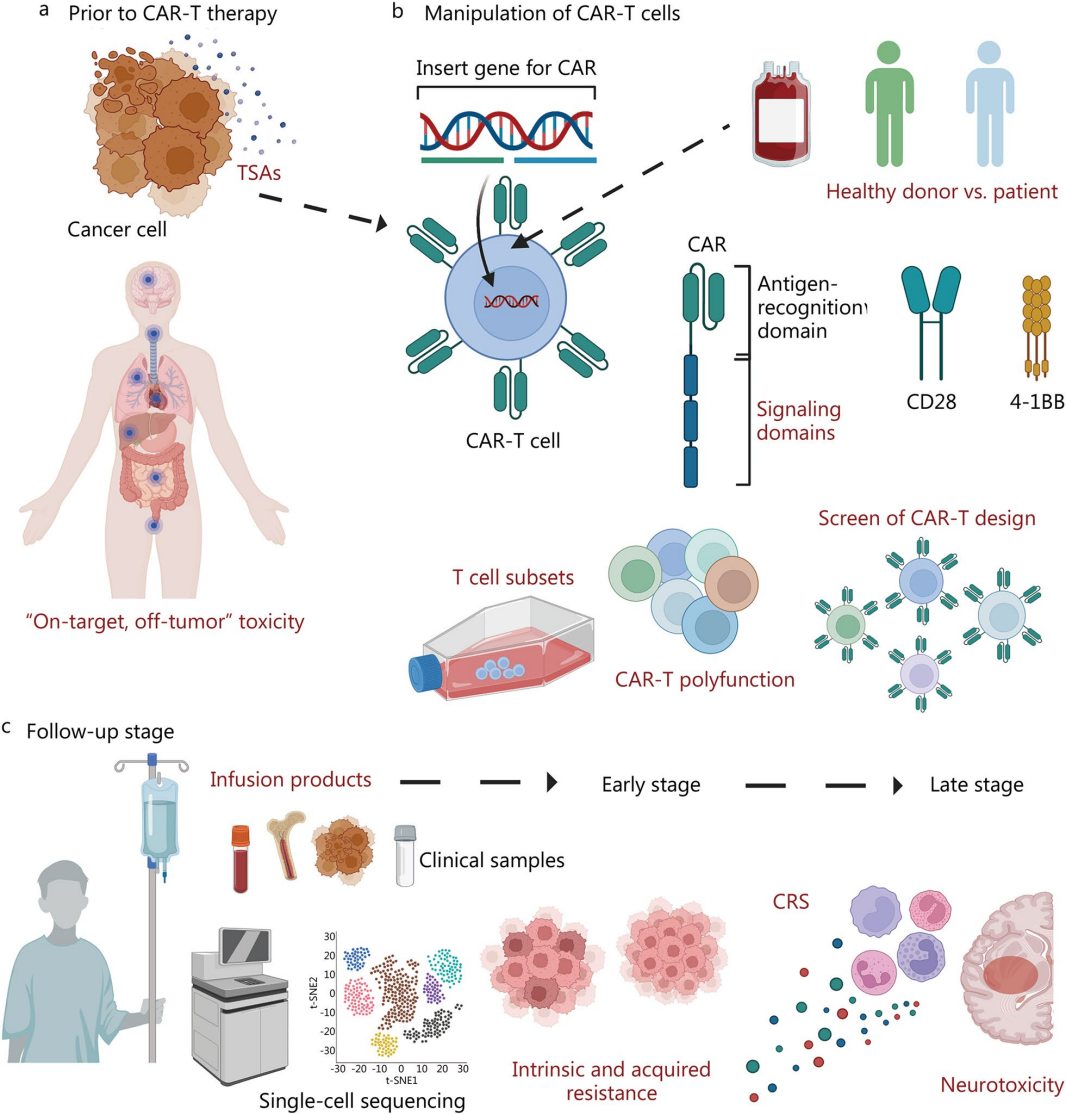
Single-cell sequencing will affect the future studies of CAR-T therapy. For every stage of CAR-T cell therapy, many essential issues require deeper interrogation. Single-cell approaches may be well-positioned to provide this information and affect the future of CAR-T therapy. **a** Prior to CAR-T therapy, **b** Manipulation of CAR-T cells, **c** Follow-up of CAR-T therapy, CAR-T chimeric antigen receptor T, TSAs tumor-specific antigens, CRS cytokine release syndrome

Although great strides have been made in CAR-T cell therapy, much more information is needed before it can be applied to a broader range of cancers. An increasing number of patients with various cancers are expected to receive cell therapy. Using patient samples, single-cell technologies will undoubtedly answer essential questions about the relationships among cancer cells, CAR-T cells and other endogenous immune populations. At each stage of CAR-T cell therapy, many fundamental questions need to be addressed in depth. Single-cell approaches may be well positioned to provide this information and shape the future of CAR-T therapy (Fig. 4).

### Prior to CAR-T therapy

Despite therapeutics targeting multiple antigens, CAR-T cell therapy has led to relatively limited success beyond hematological malignancies. The heterogeneity of various cancers, especially solid cancers, plays a crucial role in the development and progression of the disease [262, 263]. The clonal diversity of complex cancers is essential for the selection of appropriate TSA candidates, which is especially important when dealing with heterogeneous tumors [264]. The emerging single-cell technologies have the potential to provide new insights into tumor heterogeneity and to elucidate the mechanisms of immune response and immunotherapy. More importantly, the expression of CAR-target antigens in normal organs and tissues leads to the risk of “on-target, off-tumor” targeting. To better understand the effect of CAR-directed immunotherapy, a systematic single-cell-level dissection of the expression of divergent CAR-targets in various cell types across different normal tissues is also urgently required. Single-cell genomic technologies may help scholars explore potential TSAs and reflect on the rationale for antigen targets currently used in CAR-T immunotherapies and their adverse effects to minimize “off-target” immune cell activation in clinical practice. Moreover, the characterization of TME before CAR-T cell therapy can also help to predict the sensitivity and long-term response.

### Manipulation of CAR-T cells

It remains a challenge to understand what contributes to the variability of CAR-T cell products and how to make a successful CAR-T cell product. Single-cell sequencing provides a powerful and reliable tool to comprehensively describe CAR-T cells and environmental immune cell subsets. It provides a new approach to identifying immune signatures and supports the personalization of therapeutic strategies to refine CAR-T cell therapy. In this way, CAR-T cells could be appropriately manipulated for better therapeutic outcomes based on single-cell analysis. For example, engineered T cells from different individuals may be intrinsically different, resulting in different clinical outcomes. How the intrinsic T cell heterogeneity mediates therapeutic efficacy and whether allogeneic CAR-T cells are as effective as autologous cell products have been discussed in the previous sections [35]. Thus, single-cell approaches allow further exploration of the heterogeneity of donor and patient CAR-T cells, providing a novel mechanistic basis for improving clinical efficacy. The co-stimulatory domain also demonstrated the heterogeneity of unique T cell profiles and their impact on clinical outcomes [219].

T cells express a broad spectrum of immune programs, but it is unclear which programs are explicitly expressed by CAR-T cells and how the polyfunctional CAR-T cells operate. Previous studies have proven that CD4^+^ and CD8^+^ T cells comprise functionally and transcriptionally distinct subsets that differ in their capacities to proliferate, anti-tumor effects as well as persistence in vivo [265, 266]. In addition, clonally derived CD8^+^ T cells isolated from central memory T cells are distinct from those derived from effector memory T cells and retain an intrinsic capacity that permits them to survive after adoptive transfer and revert to the memory cell pool [250, 265]. Long-lived, self-renewing, multipotent T memory stem cells (T_SCM_) can trigger profound and sustained tumor regression [267]. Therefore, CAR-T infusion products were heterogeneous T cell subsets at the early stage and may lead to varying treatment outcomes [250, 267, 268]. What constitutes the optimal T cell phenotype for adoptive cell therapies remains an elusive issue, and selecting T cells for expansion or engineering is an essential step. Single-cell sequencing has shown the potential to find CAR-engineered T cell activation as a canonical process [236], which could provide a promise for CAR-T optimization.

Furthermore, single-cell sequencing can enable high-throughput screening for CAR-T cell phenotypes associated with therapeutic efficacy. CRISPR knock-in targeting can improve cell therapies, but it is unclear which knock-in gene constructs most potently enhance primary cell functions in vivo, and more high-throughput methods are required [269]. CRISPR-based functional studies in T cells could prioritize novel targets and improve the design of genetically reprogrammed cell-based therapies [270]. Shifrut et al. [271] used scRNA-seq coupled to CRISPR/Cas9 genome editing to screen gene knockout phenotypes in primary T cells. As single-cell sequencing tools can be used to resolve complex cell populations, simultaneous screening of diverse CAR designs is also an exciting prospect. Altogether, single-cell methods lay the foundation for the customized CAR designs for specific patients and offer a path toward the next step in personalized and precision medicine.

### Follow-up of CAR-T therapy

Although CAR-T cell effectiveness has been evaluated in both animal models and early-phase clinical trials with encouraging preliminary results, many other uncontrollable factors might interfere with the treatment effectiveness in cancer patients. For example, little is known about how the clonal composition of CAR-T cells changes in the recipient after adoptive transfer and how distinct transcriptional signatures in the CAR-T cells might influence cell fate in vivo. Moreover, the questions remain as to which CAR-T cells survive over time in the human body post-infusion and which factors may contribute to the responses and toxicities associated with CAR-T therapies. Tracing the TCR clonal composition of patient-retrieved CAR-T cells has made it possible to assess an increase or decrease in the relative frequencies of single gene-edited T cells following treatment. Multi-omics single-cell analysis revealed clonal expansion, proliferation, and activation in long-persisting CAR-T cells from one patient nine years after CAR-T infusion [191]. The single-cell transcriptomic and proteomic landscape also unveiled cellular and molecular mechanisms that were correlated with response to CAR-T therapy and may inform strategies to boost specific CAR-T cell function to maintain long-term remission [35, 224, 225, 238]. More importantly, single-cell sequencing can be used for the comprehensive characterization of cellular subpopulations and molecular details as well as their relations to adverse effects, which may help to overcome clinical toxicities in the clinic [41, 125, 227]. These studies highlight the utility of single-cell approaches in the study of molecular profiles linked to clinical outcomes through high-resolution views. More broadly, the aforementioned findings demonstrated the value of single-cell approaches in the generation of essential information that can serve as feedback to clinical practice in future studies.

## What the challenges lie ahead?

Although single-cell techniques have been widely adopted due to the advances in robust experimental protocols and increasing consensus surrounding quality control and data analysis, several issues still impede their widespread application in CAR-T therapies, and such challenges will dictate the development of the entire field of T cell engineering in the future. Many notable challenges and limitations have been indicated on the basis of multiple perspectives. The following as some limitations and hurdles we should take into account when performing single-cell sequencing.

Due to the reliance on high sequencing costs, advanced computational tools, and technical expertise, various single-cell technologies have not been widely applied, particularly in clinical monitoring following CAR-T therapy. Among all platforms, 10 × Genomics is the most commonly used platform for single-cell sequencing research and by most technology companies, which gives us a lot of experience and contributes to our research. Nevertheless, all platforms face the problem of high costs. Single-cell sequencing is still not an economical alternative method, which means that we cannot detect many samples, especially in multi-omics [272, 273]. It can only be used for novel discovery but not for large-scale validation. Therefore, single-cell sequencing is always compromised with bulk sequencing to make the studies more reasonable and convincing [274, 275]. In addition, to better optimize CAR-T cell products, the biological properties of T cells themselves, which are the basis of CAR-T therapies, should be further understood by using single-cell sequencing. Although we have gained a lot of insight using bioinformatics technology, the technology is only a tool to discover, and most of the findings require to be experimentally validated for reliability. Therefore, single-cell sequencing still needs to be combined with experimental validation to provide more credible results before it can be applied to the clinic.

Technologically, single-cell sequencing is subject to many limitations. The droplet-based 10 × Genomics is more likely to produce bias from doublets, while with the microwell-based platform, doublets could be avoided to the greatest extent because the depth and size of microwells are more properly designed [142]. Single-cell sequencing also has strict requirements for samples and requires a large number of cells. Most single-cell technologies are used mainly for fresh tissue samples, not for tissues that are difficult to separate or freeze. The dissociation efficiency of various tissue samples is different, and insufficient cell dissociation may result in the failure to harvest all types of cells in the tissue. Alternatively, many methods require minimal sample processing, thereby preserving cell viability and endogenous gene expression patterns [140]. Therefore, researchers should well plan and tailor the preparation process of samples [187]. Although the information obtained from single-nucleus sequencing does not match that obtained from single-cell sequencing, single-nucleus sequencing is recommended to be performed for samples that have been frozen for too long or are in a low-activity state [276]. Nonetheless, single-nucleus sequencing is usually conducted on the tissues (including brain and cardioid) [277, 278], and seems not suitable for immune cells, especially CAR-T cells. In addition, for some large tissues (such as bone), sample preparation is difficult. Another limitation of single-cell sequencing platforms is that researchers need to distinguish sequencing between 3′-ends and 5′-ends. The 3′-ends RNA sequencing is suitable for research that only requires scRNA-seq, while 5′-ends RNA sequencing provides more information and can meet the needs of TCR/BCR V-D-J sequencing [279]. Therefore, researchers need to choose specific single-cell sequencing technology according to their own research purposes and needs.

Furthermore, sequencing results also reveal the limitations of different platforms. On single-cell sequencing platforms, only a small fraction of cells from bulk tissues can be sequenced, making it difficult to discover unique and rare cell clusters, and the distribution of cells throughout the entire tissue is not yet clear [280]. To more precisely understand the information of the microenvironment and target cells, fluorescence-activated cell sorting (FACS) is often used [258, 281, 282]. Another problem is that the cell capture ability differs among platforms. For instance, neutrophils were not detected in most previous single-cell studies, with only one exception on human pancreatic ductal adenocarcinoma using 10 × Genomics Chromium platform [283]. On the other hand, BD Rhapsody can quantitatively determine the expression profiles of neutrophils [275, 284]. Similar problems also exist with the data of captured genes. Compared with 10 × Genomics, Smart-seq2 produces lower noise for mRNAs with low expressions and can be used to detect more genes in each individual cell, especially low-abundance transcript variants and alternative splicing transcripts, but capture a higher proportion of mitochondrial genes [285]. However, spatial information of individual cells in the tissue is often lost during the isolation. Therefore, spatial transcriptomics should be taken into account in research [286, 287]. Lastly, although the most commonly used scRNA-seq technology can discover novel cell types and their states in heterogeneous tissues, it is challenging to distinguish immune cells with similar transcriptomes but different functions. Since some primary sources of cellular heterogeneity may not be closely related to the transcriptome, sequencing information acquired from the transcriptome alone cannot accurately define the cellular state. There are many popular combinations for developing multimodal methods, such as spatial omics and mRNA transcriptomic e.g. cycling single-molecule fluorescence in situ hybridization (osmFISH) [288], multiplexed error robust fluorescence in situ hybridization (MERFISH) [289], sequential fluorescence in situ hybridization (seqFISH) [290], spatially resolved transcript amplicon readout mapping (STARmap) [291] and DNA methylation and chromatin accessibility (e.g. single-cell Nucleosome Occupancy and Methylome-sequencing, scNOMe-seq). Altogether, integrating different types of data is essential, and the demand for single-cell multi-omics technology is also increasing.

## Conclusions

The past decade has witnessed dramatic advances in CAR-T cell immunotherapy in the field of cancer treatment. Future development of CAR-T therapy will hinge on our growing understanding of the behavior of engineered T cells. Illustrating the phenotype of individual CAR-T cells and dynamics throughout clinical treatment is pivotal to the understanding of antitumor immunity. Single-cell profiling technologies have highlighted immense power, particularly through the study of molecular characteristics of individual cells that are undetectable by conventional bulk sequencing approaches. It is increasingly evident that single-cell methods can help reveal the optimum receptor design, guide gene-based T cell modification, and optimize CAR-T manufacturing conditions. Moreover, the detailed information generated by single-cell sequencing can not only provide insights into the numerous complex determinants of therapeutic efficacy and toxicities but also help identify predictive biomarkers and work out novel therapeutic strategies. Given that future studies will help address these challenges, single-cell sequencing technologies may eventually become a standard and essential tool for the study of CAR-T therapy. The refinements and innovations of single-cell-omics approaches, on the strength of their high resolution, will reveal the relationships between critical drivers of cancer phenotypes and therapeutic outcomes of CAR-T therapy, thereby leading to more comprehensive delivery of authentic personalized treatment for cancer patients.

## Data Availability

Not applicable.

## Abbreviations

AHCA: Adult human cell atlas
AML: Acute myeloid leukemia
axi-cel: Axicabtagen ciloleucel
B-ALL: B-cell acute lymphoblastic leukemia
BATF: Basic leucine zipper ATF-like transcription factor
BCMA: B cell maturation antigen
BCR: B cell receptor
BLIMP1: B lymphocyte-induced maturation protein 1
BM: Blood marrow
CAR: Chimeric antigen receptor
CAR-Treg: CAR-T regulatory
CD86: Cluster of differentiation 86
CEL-seq: Cell expression by linear amplification and sequencing
CITE-seq: Cellular indexing of transcriptomes and epitopes by sequencing
CNVs: Copy number variations
CRISPR: Clustered regularly interspaced short palindromic repeats
CRS: Cytokine release syndrome
CSF1R: Colony-stimulating factor 1 receptor
CyTOF: Mass cytometry by time-of-flight
FACS: Fluorescence activating cell sorter
FDA: Food and Drug Administration
GEO: Gene Expression Omnibus
HCL: Human Cell Landscape
ICANS: Immune effector cell-associated neurotoxicity syndrome
IL-6: Interleukin-6
IFN-γ: Interferon-γ
IRF4: Interferon regulatory factor 4
IRF7: Interferon regulator factor 7
LBCL: Large B-cell lymphoma
MARS-seq: Massively parallel RNA single-cell sequencing
MERFISH: Multiplexed error robust fluorescence in situ hybridization
MULTI-seq: Multiplexing using lipid-tagged indices for single-cell and single-nucleus RNA sequencing
NHL: Non-Hodgkin lymphoma
NR4A3: Nuclear receptor subfamily 4 group A member 3
osmFISH: Cycling single-molecule fluorescence in situ hybridization
PB: Peripheral blood
PBMC: Peripheral blood mononuclear cell
PCR: Polymerase chain reaction
PD-1: Programmed death-1
R2C2: Rolling circle amplification to concatemeric consensus
REAP-seq: RNA expression and protein sequencing
scATAC-seq: Single-cell assay for transposase-accessible chromatin using sequencing
scBS-seq: Single-cell bisulfite sequencing
scCGI-seq: Genome-wide CpG island methylation sequencing for single cells
scChIP-seq: Single-cell chromatin immunoprecipitation followed by sequencing
scCUT&Tag: Single-cell Cleavage Under Targets and Tagmentation
scFTD-seq: Single-cell freeze–thaw lysis directly toward 3′ mRNA sequencing
scFv: Single chain fragment variable
scHi-C-seq: Single-cell Hi-C method for chromosome conformation
sciATAC-seq: Single-cell combinatorial indexing assay for transposase-accessible chromatin using sequencing
sci-MET: Single-cell combinatorial indexing for methylation analysis
sci-Plex: Single-cell combinatorial indexing for multiplex transcriptomics
sci-RNA-seq: Single-cell combinatorial indexing RNA sequencing
SCI-seq: Single-cell combinatorial indexed sequencing
scISOr-seq: Single-cell isoform RNA sequencing
SCITO-seq: Single-cell combinatorial indexed cytometry sequencing
scNanoATAC-seq: Single-cell assay for transposase-accessible chromatin on Nanopore sequencing platform
ScNaUmi-seq: Single-cell nanopore sequencing with UMIs
scNOMe-seq: Single-cell nucleosome occupancy and methylome-sequencing
scRRBS: Single-cell reduced representation bisulfite sequencing
scTHS-seq: Single-cell transposome hypersensitive site sequencing
scWGS: Single-cell whole genome sequencing
seqFISH: Sequential fluorescence in situ hybridization
SMART-seq: Switching mechanism at 5′ end of the RNA transcript sequencing
SMOOTH-seq: Single-molecule real-time sequencing of long fragments amplified through transposon insertion
snmC-seq: Single nucleus methylcytosine sequencing
SNS: Single nuclear sequencing
SNVs: Single nucleotide variants
SPLiT-seq: Split-pool ligation-based transcriptome sequencing
STARmap: Spatially resolved transcript amplicon readout mapping
STAT3: Signal transducer and activator of transcription 3
SVs: Structure variations
TCF: Transcription factor
TCR: T cell receptor
TF: Transcription factors
Th1: T helper 1
TIMING: Time-lapse imaging microscopy in nanowell grids
TME: Tumor microenvironment
TSAs: Tumor-specific antigens
UMIs: Unique molecular identifiers
